# Supplementary material for: Socioeconomic differentials in hypertension based on JNC7 and ACC/AHA 2017 guidelines mediated by body mass index: Evidence from Nepal demographic and health survey
Source: PLoS One. 2020 Jan 27;15(1):e0218767. doi: 10.1371/journal.pone.0218767 (PMC6984730; doi:10.1371/journal.pone.0218767)
Supplement: S1 File — (DOCX) [file pone.0218767.s001.docx]

# SUPPLEMENTARY MATERIAL

**Socioeconomic Differentials in Hypertension based on JNC7 and ACC/AHA 2017 Guidelines Mediated by Body Mass Index: Evidence from Nepal Demographic and Health Survey**

Juwel Rana^1, 2,3*^; Zobayer Ahmmad^4^; Kanchan Kumar Sen^5^; Sanjeev Bista^6^; and Rakibul M Islam^7^

# Supplementary Tables

# S1 Table: Association of blood pressure outcome with education levels stratified by sex in Nepal

| **Predictor** | **Overall** | **Men** | **Women** |
| --- | --- | --- | --- |
|  | **Adjusted Odds ratios (95 % CI)** | **Adjusted Odds ratios (95 % CI)** | **Adjusted Odds ratios (95 % CI)** |
| **Hypertension (medical)** | | | |
| **Education Levels (Ref = No education/preschool)** | | | |
| Primary | 1.57 (1.32, 1.87)*** | 1.73 (1.34, 2.22)*** | 1.55 (1.24, 1.94)*** |
| Secondary | 1.92 (1.64, 2.25)*** | 2.34 (1.82, 3.02)*** | 1.48 (1.18, 1.89)** |
| Higher | 2.00 (1.59, 2.52)*** | 2.38 (1.75, 3.23)*** | 1.63 (1.18, 2.25)** |
| **Normal Blood Pressure (medical)** | | | |
| Primary | 0.68 (0.60, 0.78)*** | 0.61 (0.48, 0.77)*** | 0.81 (0.67, 0.98)* |
| Secondary | 0.58 (0.51, 0.66)*** | 0.50 (0.40, 0.62)*** | 0.76 (0.64, 0.92)** |
| Higher | 0.61 (0.51, 0.72)*** | 0.48 (0.37, 0.64)*** | 0.88 (0.69, 1.11) |
| **Hypertension (measured)** | | | |
| **Education Levels (Ref = No education/preschool)** | | | |
| Primary | 1.52 (1.25, 1.85)*** | 1.69 (1.30, 2.19)*** | 1.35 (1.07, 1.70)* |
| Secondary | 1.75 (1.46, 2.10)*** | 2.00 (1.53, 2.63)*** | 1.31 (1.00, 1.71)* |
| Higher | 1.64 (1.26, 2.13)*** | 1.89 (1.36, 2.61)*** | 1.20 (0.79, 1.83) |
| **Normal Blood Pressure (measured)** | | | |
| Primary | 0.69 (0.60, 0.79)*** | 0.61 (0.48, 0.77)*** | 0.84 (0.70, 1.01) |
| Secondary | 0.60 (0.53, 0.69)*** | 0.52 (0.41, 0.65)*** | 0.83 (69, 1.00) |
| Higher | 0.66 (0.56, 0.77)*** | 0.51 (0.40, 0.67)*** | 1.04 (0.83, 1.29) |

(Odds ratios are adjusted for age, urbanity and marital status; * p<0.05, ** p<0.01, *** p<0.001.)

**S2 Table: Association of blood pressure outcome with wealth quintiles stratified by sex in Nepal**

| **Predictors** | **Overall** | | | **Men** | **Women** |
| --- | --- | --- | --- | --- | --- |
|  | **Adjusted Odds ratios (95 % CI)** | | | **Adjusted Odds ratios (95 % CI)** | **Adjusted Odds ratios (95 % CI)** |
| **Hypertension (medical)** | | | | | |
| **Wealth Quintiles (Ref = Poorest)** | | | | | |
| Poorer | 1.28 (1.08, 1.52)* | | | 1.31 (1.04, 1.64)* | 1.28 (1.00, 1.61)* |
| Middle | 1.15 (0.94, 1.39) | | | 1.00 (0.78, 1.29) | 1.30 (1.00, 1.67)* |
| Richer | 1.23 (1.02, 1.49)* | | | 1.19 (0.93, 1.53) | 1.26 (0.98, 1.61) |
| Richest | 2.33 (1.90, 2.86)*** | | | 2.13 (1.60, 2.85)*** | 2.54 (2.00, 3.24)*** |
| **Normal Blood Pressure (medical)** | | | | | |
| Poorer | 1.00 (0.85, 1.19) | | | 1.09 (0.87, 1.35) | 0.93 (0.77, 1.13) |
| Middle | 1.17 (0.98, 1.40) | | | 1.32 (1.05, 1.66)* | 1.06 (0.86, 1.31) |
| Richer | 1.08 (0.89, 1.30) | | | 1.06 (0.84, 1.36) | 1.13 (0.91, 1.41) |
| Richest | 0.72 (0.60, 0.87)** | | | 0.74 (0.57, 0.96)* | 0.71 (0.57, 0.88)** |
| **Hypertension (measured)** | | | | | |
| **Wealth Quintiles (Ref = Poorest)** | | | | | |
| Poorer | | 1.22 (1.02, 1.46)* | 1.28 (1.01, 1.62)* | | 1.18 (0.92, 1.51) |
| Middle | | 0.99 (0.80, 1.23) | 0.92 (0.70, 1.21) | | 1.05 (0.80, 1.39) |
| Richer | | 1.05 (0.85, 1.29) | 1.04 (0.79, 1.40) | | 1.03 (0.78, 1.36) |
| Richest | | 1.64 (1.30, 2.05)*** | 1.66 (1.26, 2.19)*** | | 1.60 (1.20, 2.12)** |
| **Normal Blood Pressure (measured)** | | | | | |
| Poorer | | 1.01 (0.81, 1.19) | 1.08 (0.91, 1.35) | | 0.94 (0.77, 1.13) |
| Middle | | 1.22 (1.02, 1.47)* | 1.34 (1.06, 1.70)* | | 1.14 (0.92, 1.41) |
| Richer | | 1.11 (0.92, 1.34) | 1.10 (0.86, 1.38) | | 1.16 (0.93, 1.45) |
| Richest | | 0.81 (0.67, 0.98)* | 0.77 (0.60, 0.99)* | | 0.85 (0.67, 1.07) |

(Odds ratios are adjusted for age, urbanity and marital status; * p<0.05, ** p<0.01, *** p<0.001.)

**S3 Table: Association of blood pressure outcome with education levels stratified by urbanity in Nepal**

| **Predictors** | **Overall** | | **Urban** | | **Rural** |
| --- | --- | --- | --- | --- | --- |
|  | **Adjusted Odds ratios (95 % CI)** | | **Adjusted Odds ratios (95 % CI)** | | **Adjusted Odds ratios (95 % CI)** |
| **Hypertension (medical)** | | | | | |
| **Education Levels (Ref = No education/preschool)** | | | | | |
| Primary | 1.54 (1.29, 1.84)*** | | 1.48 (1.17, 1.86)** | | 1.51 (1.16, 1.98)** |
| Secondary | 1.91 (1.62, 2.26)*** | | 1.74 (1.42, 2.13)*** | | 1.98 (1.49, 2.64)*** |
| Higher | 2.04 (1.62, 2.57)*** | | 1.91 (1.43, 2.56)*** | | 1.86 (1.27, 2.72)** |
| **Normal Blood Pressure (medical)** | | | | | |
| Primary | 0.78 (0.68, 0.90)** | | 0.89 (0.74, 1.03) | | 0.70 (0.56, 0.87)** |
| Secondary | 0.68 (0.58, 0.82)*** | | 0.72 (0.60, 0.85)*** | | 0.61 (0.55, 0.82)*** |
| Higher | 0.66 (0.65, 0.66)*** | | 0.73 (0.58, 0.93)* | | 0.65 (0.47, 0.90)* |
| **Hypertension (measured)** | | | | | |
| **Education Levels (Ref = No education/preschool)** | | | | | |
| Primary | | 1.43 (1.18, 1.72)*** | | 1.31 (1.03, 1.67)* | 1.54 (1.15, 2.08)* |
| Secondary | | 1.65 (1.36, 1.99)*** | | 1.41 (1.12, 1.79)** | 1.97 (1.42, 2.73)*** |
| Higher | | 1.57 (1.21, 2.03)** | | 1.39 (1.00, 1.94)* | 1.70 (1.16, 2.49)** |
| **Normal Blood Pressure (measured)** | | | | | |
| Primary | | 0.80 (0.69, 0.92)** | | 0.93 (0.78, 1.11) | 0.68 (0.53, 0.88)** |
| Secondary | | 0.70 (0.61, 0.82)*** | | 0.79 (0.67, 0.95)* | 0.64 (0.50, 0.82)*** |
| Higher | | 0.76 (0.63, 0.90)** | | 0.83 (0.67, 1.03) | 0.69 (0.51, 0.94)* |

(Odds ratios are adjusted for age, sex and marital status; * p<0.05, ** p<0.01, *** p<0.001.)

**S4 Table: Association of blood pressure outcome with wealth quintiles stratified by urbanity in Nepal**

| **Predictors** | | **Overall** | | **Urban** | **Rural** |
| --- | --- | --- | --- | --- | --- |
|  |  | **Adjusted Odds ratios (95 % CI)** | | **Adjusted Odds ratios (95 % CI)** | **Adjusted Odds ratios (95 % CI)** |
| **Hypertension (Medical)** | | | | | |
| **Wealth Quintiles (Ref = Poorest)** | | | | | |
| Poorer | | 1.32 (1.11, 1.56)** | | 1.10 (0.84, 1.44) | 1.51 (1.20, 1.90)** |
| Middle | | 1.18 (0.97, 1.43) | | 1.15 (0.87, 1.51) | 1.13 (0.84, 1.52) |
| Richer | | 1.28 (1.06, 1.53)* | | 1.36 (1.05, 1.76)* | 1.01 (0.74, 1.38) |
| Richest | | 2.51 (2.06, 3.05)*** | | 2.34 (1.79, 3.08)*** | 2.35 (1.54, 3.58) |
| **Normal Blood Pressure (medical)** | | | | | |
| Poorer | | 0.98 (0.82, 1.15) | | 1.04 (0.84, 1.30) | 0.92 (0.71, 1.19) |
| Middle | | 1.14 (0.96, 1.36) | | 1.08 (0.86, 1.37) | 1.23 (0.96 (1.59) |
| Richer | | 1.05 (0.87, 1.26) | | 0.92 (0.72, 1.16) | 1.36 (1.01, 1.82)* |
| Richest | | 0.67 (0.57, 0.80) | | 0.67 (0.53, 0.84)** | 0.75 (0.51, 1.10) |
| **Hypertension (Measured)** | | | | | |
| **Wealth Quintiles (Ref = Poorest)** | | | | | |
| Poorer | 1.25 (1.05, 1.49)* | | 1.10 (0.83, 1.46) | | 1.40 (1.09, 1.78)** |
| Middle | 1.00 (0.81, 1.25) | | 1.05 (0.79, 1.40) | | 0.92 (0.64, 1.31) |
| Richer | 1.07 (0.87, 1.32) | | 1.20 (0.90, 1.60) | | 0.83 (0.59, 1.15) |
| Richest | 1.73 (1.40, 2.15) | | 1.65 (1.25, 2.19)** | | 1.84 (1.14, 2.97)* |
| **Normal Blood Pressure (measured)** | | | | | |
| Poorer | 0.98 (0.83, 1.15) | | 1.02 (0.83, 1.26) | | 0.94 (0.73, 1.20) |
| Middle | 1.19 (0.99, 1.42) | | 1.09 (0.86, 1.38) | | 1.34 (1.01, 1.77)* |
| Richer | 1.08 (0.90, 1.30) | | 0.93 (0.73, 1.19) | | 1.39 (1.04, 1.86)* |
| Richest | 0.76 (0.63, 0.91)** | | 0.74 (0.59, 0.93)* | | 0.82 (0.55, 1.25) |

(Odds ratios are adjusted for age, sex and marital status; * p<0.05, ** p<0.01, *** p<0.001.)

**S5 Table: Association of overweight/obesity (South Asia specific definition) with education levels and wealth quintiles by urbanity in Nepal**

| **Association of Overweight/Obesity (South Asia specific definition) with education levels and wealth quintiles by urbanity in Nepal** | | | |
| --- | --- | --- | --- |
| **Predictors** | **Overall** | **Urban** | **Rural** |
|  | **Adjusted Odds ratios (95 % CI)** | **Adjusted Odds ratios (95 % CI)** | **Adjusted Odds ratios (95 % CI)** |
| **Model 1: Association of Overweight-SA with Education Level**  (Odds are adjusted for age, marital status and urbanity; * p<0.05, ** p<0.01, *** p<0.001) | | | |
| **Education (Ref = No education/preschool)** | | | |
| Primary | 1.98 (1.74, 2.27)*** | 2.00 (1.68, 2.38)*** | 1.69 (1.38, 2.01)*** |
| Secondary | 2.84 (2.46, 3.27)*** | 2.73 (2.28, 3.29)*** | 2.37 (1.90, 2.97)*** |
| Higher | 4.16 (3.48, 4.97)*** | 4.09 (3.24, 5.17)*** | 2.90 (2.22, 2.80)*** |
| **Model 2: Association of Overweight-SA with Wealth Quintiles**  (Odds are adjusted for age, marital status and urbanity; * p<0.05, ** p<0.01, *** p<0.001) | | | |
| **Wealth Quintiles (Ref = Poorest)** | | | |
| Poorer | 1.48 (1.24, 1.77)*** | 1.43 (1.09, 1.89)* | 1.54 (1.20, 1.97)** |
| Middle | 1.63 (1.36, 1.96)*** | 1.81 (1.35, 2.43)*** | 1.43 (1.10, 1.86)** |
| Richer | 2.59 (2.15, 3.12)*** | 2.96 (2.22, 2.95)*** | 2.07 (1.60, 2.66)*** |
| Richest | 7.22 (6.03, 8.65)*** | 8.16 (6.19, 10.76)*** | 3.66 (2.30, 5.80)*** |

**S6 Table: Linear Regression comparing BMI with educational levels and wealth quintiles in Nepal**

| **Association between BMI and Socioeconomic Status in Nepal** | |
| --- | --- |
| **Predictors** | **Adjusted coefficient**  **(95 % CI)** |
| **Model 1: Association between BMI and Education Levels**  (Coefficients are adjusted for age, marital status and urbanity; * p<0.05, ** p<0.01, *** p<0.001) | |
| **Education (Ref = No education/preschool)** | |
| Primary | 1.25 (1.01, 1.49)*** |
| Secondary | 1.90 (1.64, 2.18)*** |
| Higher | 2.47 (2.13, 2.81)*** |
| **Model 2: Association between BMI and Wealth Quintiles**  (Coefficients are adjusted for age, marital status and urbanity; * p<0.05, ** p<0.01, *** p<0.001) | |
| **Wealth (Ref = Poorest)** | |
| Poorer | 0.30 (0.03, 0.57)* |
| Middle | 0.39 (0.09, 0.68)* |
| Richer | 1.41 (1.08, 1.74)*** |
| Richest | 3.67 (3.29, 4.05)*** |

**Table S7:** **Mediation effect (by 10% change in coefficients after adjusting for mediator) of BMI on SES and hypertension by sex in Nepal**

| **Predictor** | **Model I- Overall (n=13,436)** | | **Model II-Men (n=5,646)** | | **Model III-Women (n=7,790)** | |
| --- | --- | --- | --- | --- | --- | --- |
|  | **^a^Regression Coefficients without mediator (95 % CI)** | **^b^Mediator Adjusted Regression Coefficients (95 % CI)** | **^a^Regression Coefficients without mediator (95 % CI)** | **^b^Mediator Adjusted Regression Coefficients (95 % CI)** | **^a^Regression Coefficients without mediator**  **(95 % CI)** | **^b^Mediator Adjusted Regression Coefficients**  **(95 % CI)** |
| **Hypertension (medical) by Education Levels (Ref. No education/preschool)** | | | | | | |
| Primary | 0.40 (0.22,0.58)*** | 0.24  (0.57,0.42)** | 0.55  (0.30,0.81)*** | 0.41  (0.16,0.66)** | 0.42  (0.19,0.64)*** | 0.23  (0.00,0.46)* |
| Secondary | 0.54  (0.42,0.75)*** | 0.35  (0.17,0.53)*** | 0.87  (0.61,1.12)*** | 0.61  (0.40,0.89)*** | 0.36  (0.12,0.60)** | 0.13  (-0.11,0.37) |
| Higher | 0.61  (0.78,0.85)*** | 0.32  (0.06,0.57)* | 0.90  (0.59,1.20)*** | 0.54  (0.21,0.88)** | 0.43  (0.11,0.75)** | 0.18  (-0.17,0.53) |
| **Hypertension (medical) by Wealth Quintiles (Ref. Poorest)** | | | | | | |
| Poorer | 0.25  (0.08,0.42)** | 0.21  (0.04,0.38) | 0.28  (0.05,0.51)* | 0.23  (-0.00,0.46)* | 0.23  (-0.01,0.46) | 0.20  (-0.04,0.43) |
| Middle | 0.13  (-0.07,0.33) | 0.08  (-0.10,0.28) | 0.02  (-0.23,0.28) | 0.05  (-0.31,0.20) | 0.23  (-0.02,0.49) | 0.19  (-0.05,0.44) |
| Richer | 0.21  (0.01,0.40)* | 003.  (-0.16,0.22) | 0.20  (-0.05,0.46) | 0.12  (-0.24,0.28) | 0.19  (-0.06,0.44) | 0.00  (-0.24,0.25) |
| Richest | 0.84  (0.63,1.05)*** | 0.41  (0.18,0.63) | 0.80  (0.50,1.10)*** | 0.39  (0.03,0.75)* | 0.88  (0.64,1.12)*** | 0.39  (0.13,0.65)** |

^a^Coefficients adjusted for age, sex, marital status, urbanity, and second-hand smoking; ^b^Coefficients further adjusted for mediator-BMI. Regression coefficients; 95% confidence intervals in brackets; * p<0.05, ** p<0.01, *** p<0.001.
