# Supplementary material for: Socioeconomic differentials in hypertension based on JNC7 and ACC/AHA 2017 guidelines mediated by body mass index: Evidence from Nepal demographic and health survey
Source: PLoS One. 2020 Jan 27;15(1):e0218767. doi: 10.1371/journal.pone.0218767 (PMC6984730; doi:10.1371/journal.pone.0218767)
Supplement: S2 File — (DOCX) [file pone.0218767.s002.docx]

# SUPPLEMENTARY MATERIAL

**Socioeconomic Differentials in Hypertension based on JNC7 and ACC/AHA 2017 Guidelines Mediated by Body Mass Index: Evidence from Nepal Demographic and Health Survey**

Juwel Rana^1, 2,3*^; Zobayer Ahmmad^4^; Kanchan Kumar Sen^5^; Sanjeev Bista^6^; and Rakibul M Islam^7^

# Supplementary Figures

# S1 Fig: Flowchart of analytic sample selection process

#
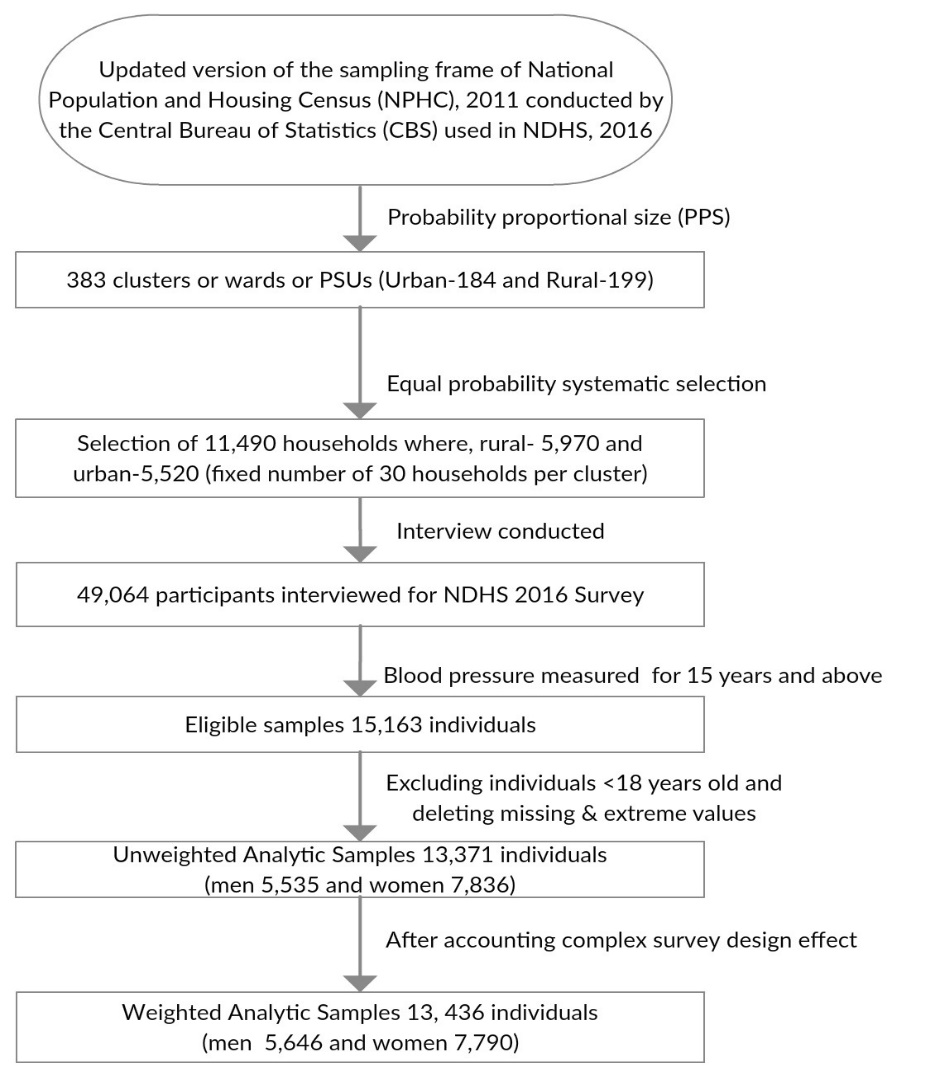


**S2 Fig: Association of hypertension (measured and medical) based on ACC/AHA 2017 guideline with education levels by sex in Nepal**


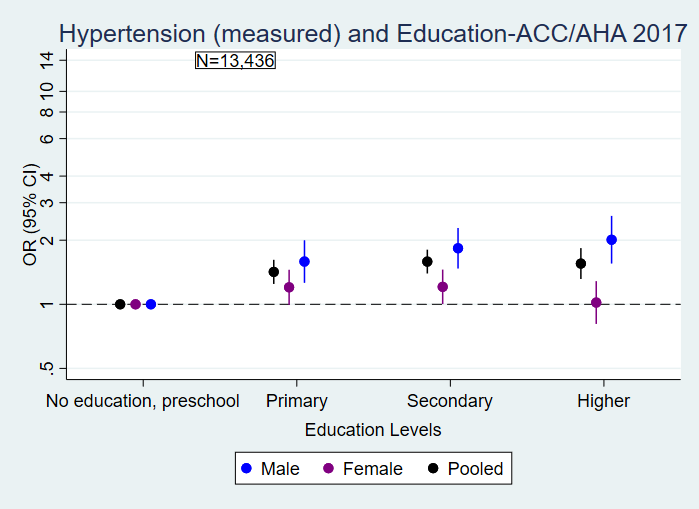


Odds ratios are adjusted for age, urbanity and marital status, and stratified by sex. Measurement-only outcomes are defined based on cut-off points only; medical outcomes are defined based on cut-off points, diagnosis by a health professional or relevant medication use.

**S3 Fig:** **Association of hypertension (measured and medical) based on by ACC/AHA 2017 guideline with wealth quintiles by sex in Nepal**


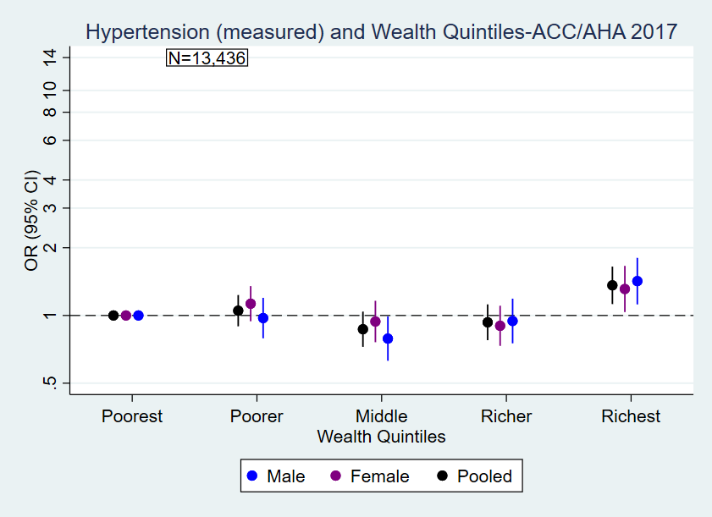


Odds ratios are adjusted for age, urbanity and marital status, and stratified by sex. Measurement-only outcomes are defined based on cut-off points only; medical outcomes are defined based on cut-off points, diagnosis by a health professional or relevant medication use.

**S4 Fig: Association of hypertension (medical) based on by ACC/AHA 2017 guideline with education levels and wealth quintiles by urbanity in Nepal**

Odds ratios are adjusted for age, sex and marital status, and stratified by urbanity. Medical outcomes are defined based on cut-off points, diagnosis by a health professional or relevant medication use.

**S5 Fig: Association of overweight/obesity (South Asia specific definition) with a) education levels b) wealth quintiles by sex in Nepal**

Odds ratios are adjusted for age, urbanity and marital status, and stratified by sex. Overweight and obese are defined as BMI ≥ 23 kg/m2 (South Asia-specific definitions).

**S6 Fig: Association of overweight/ obesity (Global definition) with a) education levels b) wealth quintiles by the sex in Nepal**

Odds ratios are adjusted for age, urbanity and marital status and stratified by sex. Overweight and obese are defined as BMI ≥ 25 kg/m2 (Global definitions).

**S7 Fig: Association of overweight/ obesity (Global definition) with a) education levels b) wealth quintiles by the urbanity in Nepal**

Odds ratios are adjusted for age, sex, and marital status and stratified by the place of residence. Overweight and obese are defined as BMI ≥ 25 kg/m2 (Global definitions).

**S8 Fig: Mediating role of BMI in the association between SES and hypertension (measured) by ACC/AHA 2017 in Nepal**

Coef. 0.15 (0.14, 0.16)^***^

Coef. 0.14 (0.13, 0.15)^***^

Coef. 4.16

(3.69, 4.64)^***^

IV: Education Levels

Direct effect- Coef. 0.63 (0.36, 0.90)^***^

MV: BMI

Indirect effect (Coef. 0.58 95% bias-corrected CI: 0.51, 0.67)^***^

MV: BMI

Indirect effect (Coef. 0.86 95% bias-corrected CI: 0.75, 0.97)^***^

DV: Hypertension (Measured)

IV: Wealth Quintiles

Direct effect- Coef. -0.44 (-0.82, -0.06)^***^

Coef. 5.86

(5.21, 6.50)^***^

Path coefficients (95% CI) and indirect effect of SES on hypertension through BMI with bias-corrected 95% confidence intervals are reported. * p<0.05, ** p<0.01, *** p<0.001. CI=Confidence Interval, BMI= Body Mass Index, DV=Dependent Variable, IV=Independent Variable, MV=Mediating Variable, SES=Socioeconomic Status.
