# Supplementary material for: Socioeconomic differentials in hypertension based on JNC7 and ACC/AHA 2017 guidelines mediated by body mass index: Evidence from Nepal demographic and health survey
Source: PLoS One. 2020 Jan 27;15(1):e0218767. doi: 10.1371/journal.pone.0218767 (PMC6984730; doi:10.1371/journal.pone.0218767)
Supplement: Attachment — Submitted filename: PONE-D-19-16119.pdf [file pone.0218767.s003.pdf]

**Referee Report**
**PONE-D-19-16119**
on
*Socioeconomic Differentials in Hypertension based on JNC7 and ACC/AHA 2017 Guidelines Mediated by Body Mass Index: Evidence from Nepal Demographic and Health Survey*

The main objective of this research is to evaluate if there is a positive association between socioeconomic status (SES) and the probability of having hypertension in Nepal. They use the 2016 Nepal Demographic and Health Survey to estimate logit models using binary dependent variable based on two definitions of blood pressure. One dependent variable is only based on the blood pressure exams taken during the interview. The second dependent variable identifies high-blood pressure individuals based on the blood pressure exams taken during the interview or on self-reported diagnosis of high pressure or medicine prescription. In addition, the authors include in the models an obesity-related variable to evaluate if the body mass index could be viewed as a mediator variable between SES and the likelihood of having high blood pressure. The authors conclude that higher SES was positively associated with the higher likelihood of having hypertension, and that such association between higher SES and hypertension was mediated by the body mass index (BMI).

The methodology used by the authors seems to be commonly used for this type of analysis but it is not properly used and described. More importantly, conclusions do not agree with the statistical result. I explain below my concerns with more detail.

# 1 Main Comments

1. The authors conclude that higher SES is positively associated with a higher likelihood of having hypertension. However this conclusion is not reliable because of two reasons:

   (a) Most of the confidence intervals (CIs) of the odd ratios (ORs) related to education or wealth overlap to each other. In particular, the confidence interval for the highest level of SES (education or wealth) is the widest. Therefore, it is possible that ORs are not statistically different across SES levels. If there is some significant difference based on the results, it would

be for any SES with respect to the lowest SES where in which it is more likely to observe under-nutrition (and probably a very low prevalence rate of obesity). These are just two examples to illustrate extreme CI overlap: (i) S1 Table, medical, women: primary education CI [1.24,1.94], higher education CI [1.18, 2.25] (ii) S2 Table, medical, overall: poorer CI [1.08,1.52], richer CI [1.02, 1.59].

(b) It is not clear how the medical high blood pressure is defined. It seems that adults that do not have high pressure based on the blood pressure readings could still be considered to have high pressure if they reported a past diagnosis of hypertension or they report that are taking some prescribed medicine for high blood pressure. Since access to health services could be correlated with higher SES, it is possible that this medical definition of high blood pressure biases the estimated relationship between SES and the probability of having high blood pressure. In fact, OR point estimates using the medical definition of high blood pressure are systematically larger than those calculated using the measured blood pressure definition, whereas their corresponding CI are always to the right of those calculated with the measured blood pressure (e.g., see Table S1-S4). This limitation of using the medical definition should be clearly stated in the manuscript.

2. Given the limitation of the medical definition of high blood pressure, the exercise of mediation presented in Table 4 should be done with the measurement-only definition. It is not clear why the authors only present the results using the medical definition. In addition, Table 4 presents results as *exponentiated coefficients* whereas the OR estimates are reported for other models. I suggest to present the results of Table 4 using the OR estimates. Since other regressors are included, exponentiated coefficients are not equal to the OR.

3. The mediator analysis is poorly explained. The authors say: *"The second step was the "indirect effect"approach, which formally examined the statistical significance of an indirect effect using the product of coefficients approach. For assessing the indirect effect of BMI on these binary outcomes, we used the binary-mediation package in Stata".* The results of this analysis are poorly reported (e.g., Figure 3 is not in the manuscript). Furthermore, to the best of my knowledge, there are at least three different methods in Stata to conduct mediation analysis. I suggest that the authors explain this procedure clearly.

4. According to Nepal DHS, 2016-Final Report, rates of hypertension are higher

among tobacco users. Therefore the mediation analysis should include this variable.

5. According to the Nepal DHS, 2016-Final Report[1] *"The average of the second and third measurements was used to classify the respondent with respect to hypertension, according to internationally recommended categories (WHO 1999; NIH 1997)"*. It is not clear why the authors use the average of the three measurements. Would the results be different if they use the variable created and suggested by the Nepal DHS? See Tables 14.3.1 and 14.3.2 to find the definitions of hypertension available in Nepal DHS. 2016.

6. It is not clear if sampling weights were included in the estimations of the logistic regressions. They should be used.

7. About policy implications. The authors suggest that interventions should be done in the higher SES groups to modify hypertension or obesity. Based on my previous comments, the results that they report contain important flaws from a methodological point of view, so they do not support this policy recommendation.

## 2 Minor Comments

1. The number of observations mentioned by the authors is smaller from the one reported in the Nepal DHS, 2016-Final Report (see Tables 14.3.1 and 14.3.2). Is it possible that the range of ages considered was only between 15-60 years old? In the case of BMI the number of observations reported in Nepal DHS seems to be different too (e.g. it was not measured for pregnant women). I suggest that the authors report the sample size for each estimated model.

2. The evidence that prevalence of hypertension is higher among high SES in low-income countries is limited to specific countries and it has been questioned[2]. Furthermore, in a recent study Rosengrent et al.(2019)[3] conclude that *"people with*

[1]Ministry of Health - MOH/Nepal, New ERA/Nepal, and ICF. 2017. Nepal Demographic and Health Survey 2016. Kathmandu, Nepal: MOH/Nepal, New ERA, and ICF. Available at http://dhsprogram.com/pubs/pdf/FR336/FR336.pdf.

[2] See Razak, F., & Subramanian, S. V. (2014). Commentary: Socioeconomic status and hypertension in low-and middle-income countries: can we learn anything from existing studies?. International journal of epidemiology, 43(5), 1577-1581.

[3] Socioeconomic status and risk of cardiovascular disease in 20 low-income, middle-income, and high-income countries: the Prospective Urban Rural Epidemiologic (PURE) study. The Lancet Global Health, 7(6), e748-e760.

*low levels of education in low-income and middle-income countries had a markedly higher risk of major cardiovascular events compared with those with higher levels of education. Cardiovascular disease in low-income countries is a problem predominantly among people with lower levels of education, whereas the situation in middle-income countries is more variable*". I suggest that the authors consider this issue at least in the discussion section.

3. Improve quality of figures.
