# Supplementary material for: Socioeconomic differentials in hypertension based on JNC7 and ACC/AHA 2017 guidelines mediated by body mass index: Evidence from Nepal demographic and health survey
Source: PLoS One. 2020 Jan 27;15(1):e0218767. doi: 10.1371/journal.pone.0218767 (PMC6984730; doi:10.1371/journal.pone.0218767)
Supplement: Attachment — Submitted filename: PONE-D-19-16119R2.pdf [file pone.0218767.s005.pdf]

Referee Report #2

PONE-D-19-16119

On

Socioeconomic Differentials in Hypertension based on JNC7 and ACC/AHA 2017 Guidelines Mediated by Body Mass Index: Evidence from Nepal Demographic and Health Survey.

I appreciate the effort of the authors to respond to all my comments. In my opinion, the quality and the clarity of the paper have improved significantly. However, I have one concern about this conclusion:

"Our study, including 13,436 people from a nationally representative survey, demonstrated that increasing levels of SES (education and wealth) were positively associated with an increased risk of having hypertension in Nepal, with an evidence of effect modification of gender in the 351 association between education and hypertension".

I disagree with this conclusion because the statistical results that they obtain do not constitute a demonstration or a proof that increasing levels of SES are positively associated with hypertension in Nepal. In fact, the statistical results present evidence that adults with some level of formal education (primary or beyond) are more likely to have hypertension in Nepal. My conclusion is based on the statistical results of Figure 1 and 2 (and the corresponding estimated models from Table 4). These figures show that the confidence intervals (CIs) of the odd ratios (ORs) related to education or wealth overlap with each other. This is the same comment that I included in my previous review. The authors' response was that "If two statistics have non-overlapping confidence intervals, they are necessarily significantly different, but if they have overlapping confidence intervals, it is not necessarily true that they are not significantly different." That is totally correct. Since there are overlapping CIs, the ORs could or could not be different. Therefore, we cannot conclude that we have demonstrated that the probability of having hypertension increases as the SES increases. This is equivalent to the result of not rejecting a null hypothesis. In that case, we would never claim that the null is true (or not true). The most precise way to solve this concern is by testing the equality of the parameters estimated in the models presented in Table 4 (without the mediator). A Wald test or a Lagrange multiplier test can be used to evaluate if the null that the parameters associated with different SES are different is rejected or not. Once again, a clear conclusion from this article is that having primary education or more increases the probability of having hypertension. As I mentioned in my previous review, this result could be explained if people with the lowest SES are also those with under-nutrition levels.

Finally, the average BMI in Nepal is around 22 with a small standard deviation. This suggests that the prevalence rate of obesity is small. How could the BMI be an important mediator for hypertension in individuals when most of them have BMI levels that are considered healthy levels? It would be worth considering this issue in the discussion section.
