# Supplementary material for: Socioeconomic differentials in hypertension based on JNC7 and ACC/AHA 2017 guidelines mediated by body mass index: Evidence from Nepal demographic and health survey
Source: PLoS One. 2020 Jan 27;15(1):e0218767. doi: 10.1371/journal.pone.0218767 (PMC6984730; doi:10.1371/journal.pone.0218767)
Supplement: Attachment — Submitted filename: Responses to Reviewers.docx [file pone.0218767.s006.docx]

20th December 2019

Bryan L. Sykes, Ph.D.

Academic Editor

PLOS ONE

RE: PONE-D-19-16119 ‘Socioeconomic differentials in hypertension based on JNC7 and ACC/AHA 2017 guidelines mediated by body mass index: Evidence from Nepal demographic and health survey.’

Dear Professor Sykes,

We thank you for the opportunity to resubmit our manuscript and for the considered comments of the reviewers. We have addressed each of these point by point below, and a revised version of the manuscript is submitted for your consideration.

We believe we have addressed all the reviewers’ comments, and where changes have been made, have shown these as marked yellow changes. We hope the manuscript is now considered acceptable for publication in your journal.

Yours sincerely,

Juwel Rana, MPH (double)
Corresponding author

**Editor’s Concern**

**First,** Reviewer 2 draws your attention to an overarching conclusion espoused in the paper. I concur with the reviewer’s assessment and request that you change the statement from “Our study, including 13,436 people from a nationally representative survey, demonstrated that increasing levels of SES (education and wealth) were positively associated with an increased risk of having hypertension in Nepal, with an evidence of effect modification of gender in the 351 association between education and hypertension” to “Our study, including 13,436 people from a nationally representative survey, finds that higher levels of education (compared to individuals with no education or at a pre-school educational level) are at an increased risk of having hypertension in Nepal, with the association moderated by gender.”

**Authors’ Response:** We appreciate your succinct summarization of reviewers’ comments. Reviewer 2’s concern is about overlapping CIs in the associations. In our first round responses, we benignly addressed the issues of CI’s overlapping. However, the concept of overlapping CI’s is only relevant when the outcome measure is continuous and normally distributed. Our outcome is binary, and effect estimates are odds ratios, which are NOT normally distributed. Like all other non-normal estimates such as risk ratio or hazard ratio, the issues of overlapping CIs cannot be applied to the odds ratio. The lower limits and upper limits of odds ratio are exponential of the margin of error of beta coefficients. Hence, the conclusion drawn from the results is valid.

Moreover, despite gender-stratified analysis, we have formally tested effect modification of gender in the associations between SES and hypertension. It reaffirms our rationale related to CI’s overlapping. Therefore, we would like to keep the statement as it currently appears in the manuscript.

However, if the editor feels the statement needs to be changed, we are happy to change it, but the statement will be partially correct based on our results.

According to editor’s suggestions, we have modified the tone of our statement in page 19, lines 352-354: “Our study, including 13,436 people from a nationally representative survey, finds that people with increasing levels of SES (education and wealth) are at an increased risk of having hypertension in Nepal, with the association (education) moderated by gender.”

**Second,** Reviewer 2 raises a question that merits engagement in your discussion section. Namely, how does BMI mediate hypertension in a population that has normal (or healthy) BMI levels?

**Authors’ Response:** We agree with this point. Due to getting low prevalence of obesity, according to the global cut-offs of BMI, we counted and categorized BMI as well as obesity/overweight according to the South Asia-specific cut-offs for BMI in our earlier version and already showed on page 19, lines 341-346 and in supplementary tables 5 and 6.

However, we have provided further clarification in the result and discussion section now according to the suggestions of the editor and reviewer in the following pages and lines:

**Result:** Page 17, lines 306-310: “The average BMI, according to the global cut-offs of BMI, was about 22, which indicates about 18% of the respondents were obese/overweight. However, the prevalence of obesity/overweight, according to the South Asia-specific cut-offs of BMI, was about 37%. The likelihood of being overweight/obese increased with an increasing level of SES, which also modified by sex and urbanity (S5 Table, S6 Fig).”

**Discussion:** Page 21, lines 385-387: “Our study demonstrated that the prevalence of obesity/overweight, according to the South Asia-specific cut-offs of BMI, was at unhealthy levels, and the risk of being obese/overweight was increased by the increasing levels of SES (education and wealth).”

**Reviewer #1:** Specific comments

Page 11: line 232 - 233; I think the reviewer should be cautious in interpreting their values since they claim there were too many missing values.

**Authors’ Response:** We completely agree with this point. This was one of the reasons not considering employment as an indicator of SES in our study. Thus, we are cautious about our interpretation and we did not include ‘employment’ as one of the main independent variables.

Table 2: sample characteristics. I suggest that the authors interpret the dummies from averages to frequencies.

Page 29: line 389 - 396 should go to the conclusions.

General

Your conclusion should address the summary, limitations and policy recommendations. Please do restructure your introduction.

**Authors’ Response:** We think now we have included more details, which addressed these comments. Moreover, our conclusion seems inclusive addressing summary, limitations and policy recommendations.

**Reviewer #2:**

A) I appreciate the effort of the authors to respond to all my comments. In my opinion, the quality and the clarity of the paper have improved significantly. However, I have one concern about this conclusion:

“Our  study,  including  13,436  people  from  a  nationally  representative  survey,  demonstrated  that  increasing levels of SES (education and wealth) were positively associated with an increased risk of having hypertension in Nepal, with an evidence of effect modification of gender in the 351 association between education and hypertension”.

I  disagree  with  this  conclusion  because  the  statistical  results  that  they  obtain  do  not  constitute  a  demonstration or a proof that increasing levels of SES are positively associated with hypertension in Nepal.

In fact, the statistical results present evidence that adults with some level of formal education (primary  or  beyond)  are  more  likely  to  have  hypertension  in  Nepal.  My  conclusion  is  based  on  the  statistical results of Figure 1 and 2 (and the corresponding estimated models from Table 4). These figures show that the confidence intervals (CIs) of the odd ratios (ORs) related to education or wealth overlap with each other. This is the same comment that I included in my previous review. The authors’ response was that “If two statistics have non‐overlapping confidence intervals, they are necessarily significantly different, but if they have overlapping confidence intervals, it is not necessarily true that they are not significantly different.” That is totally correct. Since there are overlapping CIs, the ORs could or could not be different. Therefore, we cannot conclude that we have demonstrated that the probability of having hypertension  increases  as  the  SES  increases.

This  is  equivalent  to  the  result  of  not  rejecting  a  null  hypothesis. In that case, we would never claim that the null is true (or not true). The most precise way to solve this concern is by testing the equality of the parameters estimated in the models presented in Table 4 (without the mediator). A Wald test or a Lagrange multiplier test can be used to evaluate if the null that the  parameters  associated  with  different  SES  are  different  is  rejected  or  not.  Once  again,  a  clear  conclusion from this article is that having primary education or more increases the probability of having hypertension. As I mentioned in my previous review, this result could be explained if people with the lowest SES are also those with under‐nutrition levels.

**Authors’ Response:** We have addressed this point and mentioned it in the editor’s concern.

B) Finally, the average BMI in Nepal is around 22 with a small standard deviation. This suggests that the prevalence rate of obesity is small. How could the BMI be an important mediator for hypertension in individuals when most of them have BMI levels that are considered healthy levels? It would be worth considering this issue in the discussion section.

**Authors’ Response:** We have addressed this point and mentioned it in the editor’s concern.
